# Diesel Exhaust Exposure and the Risk of Lung Cancer—A Review of the Epidemiological Evidence

**DOI:** 10.3390/ijerph110201312

**Published:** 2014-01-27

**Authors:** Yi Sun, Frank Bochmann, Annette Nold, Markus Mattenklott

**Affiliations:** Institute for Occupational Safety and Health of the German Social Accident Insurance (IFA), Alte Heerstraße 111, Sankt Augustin 53773, Germany; E-Mails: frank.bochmann@dguv.de (F.B.); annette.nold@dguv.de (A.N.); markus.mattenklott@dguv.de (M.M.)

**Keywords:** diesel exhaust, diesel motor emissions, DME, epidemiology, review, lung cancer

## Abstract

To critically evaluate the association between diesel exhaust (DE) exposure and the risk of lung cancer, we conducted a systematic review of published epidemiological evidences. To comprehensively identify original studies on the association between DE exposure and the risk of lung cancer, literature searches were performed in literature databases for the period between 1970 and 2013, including bibliographies and cross-referencing. In total, 42 cohort studies and 32 case-control studies were identified in which the association between DE exposures and lung cancer was examined. In general, previous studies suffer from a series of methodological limitations, including design, exposure assessment methods and statistical analysis used. A lack of objective exposure information appears to be the main problem in interpreting epidemiological evidence. To facilitate the interpretation and comparison of previous studies, a job-exposure matrix (JEM) of DE exposures was created based on around 4,000 historical industrial measurements. The values from the JEM were considered during interpretation and comparison of previous studies. Overall, neither cohort nor case-control studies indicate a clear exposure-response relationship between DE exposure and lung cancer. Epidemiological studies published to date do not allow a valid quantification of the association between DE and lung cancer.

## 1. Introduction

Diesel engines have been widely used for decades in various industrial sectors such as underground mining, construction, public transportation, ship loading in docks, agriculture, operation of machines and fire-fighting. Diesel exhaust (DE) emissions are composed of gases and a particulate phase containing thousands of chemicals. Their composition varies according to engine type, speed, air/fuel ratio, temperature, fuel and many other factors [1]. DE contains large quantities of carbonaceous particulates to which polynuclear aromatic hydrocarbons and other heterocyclic compounds are adsorbed. The latter are known to be mutagenic and carcinogenic in both animals and humans [2].

In June 2012, a working group of the International Agency for Research on Cancer concluded that there was sufficient evidence for the carcinogenicity of DE in humans [3]. However, these findings appear to be based upon selected epidemiological studies with certain important methodological limitations, particularly in the assessment of confounding effects and the assessment of DE exposures [4]. In order to evaluate critically the epidemiological evidence for the association between DE exposure and the risk of lung cancer, we conducted a systematic review of the international literature.

## 2. Methods

### 2.1. Literature Search

For comprehensive identification of original studies on the association between DE exposure and the incidence or mortality of lung cancer, searches were performed for the period between 1970 and 2013 in the following databases: MEDLINE, EMBASE, NIOSHTIC, CISDOC, Cochrane and the databases in TOXNET. Multipart search strategies were applied using “diesel” combined with the following search terms: “lung cancer”, “lung neoplasm?”, “work?”, “occupation?”, “epidemiol?”, “case control”, “cohort” or “risk”. Bibliographies and cross-referencing including comparison with reviews were additionally used for literature searches.

### 2.2. Quantification of DE Exposures Using MEGA-JEM

Previous studies on the effect of DE exposure focus mainly on risk estimation for jobs supposed to involve high and prolonged exposure to DE, such as those of professional drivers, railroad workers, heavy equipment operators, and so on. Although a large number of studies have been published, few are able to provide any information on the level of DE exposures in these jobs.

To allow an objective impression to be gained of the level of DE exposures in commonly exposed jobs, we created a job-exposure matrix for DE exposures based upon historical industrial hygiene data from the MEGA (Measurement data relating to workplace exposure to hazardous substances) database (see Table 1).

**Table 1:** DE exposures in common exposed jobs in Germany (MEGA-JEM).

Job Titles (MEGA job title ^(1)^)	Exposure as Elemental Carbon (mg/m^3^) ^(2)^		
	Before 1990 ^(3)^	1990–1993 ^(4)^	After 1993 ^(4)^
Dock workers,	0.19	0.05	0.03
Transportation equipment operators			
(warehouse and loading work)			
Heavy equipment operators	0.26	0.08	0.03
Drivers of heavy construction vehicles			
(shipping and transport within enterprises)			
Highway maintenance	0.13	0.04	0.02
Open-air mechanics			
Highway workers			
(repair and maintenance)			
Mechanics (not open-air)	0.18	0.09	0.03
Bus garage workers			
Truck mechanics			
(bench tests)			
Truck drivers	0.07	0.02	0.01
Heavy truck drivers			
Professional drivers			
Railroad workers			
Bus drivers			
Lorry drivers			
Taxi drivers			
(50% of exposure level of repair and maintenance)			
Potash miner	0.30	0.15	0.14

Notes: ^(1)^ Exposure data from MEGA are related to the listed job titles; ^(2)^ Exposure data are calculated from exposure data of total carbon (TC) using the known task related mean relation between EC and TC; ^(3)^ 90% percentile of the exposure data for the period 1990–1993; ^(4)^ 50% percentile of exposure data.

The MEGA database is a large industrial hygiene database forming part of the Measurement System for Exposure Assessment of the German Social Accident Insurance Institutions (MGU). The database was established in 1972 and contains more than 2.4 million historical measurements of around 1,380 industrial chemical and biological agents. In total, around 4,000 historical measurements of DE exposures were entered in the database for the period from 1990 to 2000.

In this review, MEGA-JEM was used directly to estimate the exposure levels of jobs given in the results of previous published studies. If information on exposure duration is available, cumulative doses of DE exposure were quantified as “exposure level (MEGA-JEM) × median exposure duration”. Effect estimates published in previous studies were summarized in a scatter plot. Based on these values, exposure-response relationship between DE-exposure and lung cancer and their 95% CI were quantified by a linear regression analysis with the software package SigmaPlot 12.0. The inclusion of MEGA-JEM in this review will permit a direct comparison of previously published epidemiological evidence.

## 3. Results

In total, 42 cohort studies and 32 case-control studies were identified in which the association between DE exposure and lung cancer was examined.

### 3.1. Cohort Studies

In general, historical industrial hygiene data on DE exposure (based on the measurement of elemental carbon) were not available in published cohort studies. Therefore, exposure assessment was limited only to job titles in 37 of the 42 identified cohort studies. Five studies allow a quantitative assessment of DE exposure based on industrial hygiene measurement. Three studies [5,6,7] quantified the DE exposures based upon historical surrogate measurements of nitrogen dioxide, while two other studies were based either on current industrial hygiene measurement of total carbon [8] or on historical surrogate measurements of CO [9].

The effect of DE exposure upon lung cancer was evaluated with the focus primarily on the following job categories: professional drivers, highway maintenance workers, railroad workers, mechanics, workers at gasoline filling stations, heavy equipment operators, dock workers and miners (see Table 2).

The effect of DE exposure was evaluated in most studies by comparison of the lung cancer risk among workers in highly exposed jobs with an external population by use of the standardized mortality ratio (SMR), standardized incidence ratio (SIR) or proportional mortality ratio (PMR). Internal comparison was carried out in nine cohort studies [2,5,6,7,8,9,10,11,12]. All studies have large sample sizes. The possible confounding effect of smoking was adjusted in most of these studies (except the study by Bergdahl [7] and the study by Attfield [9]).

Boffetta *et al*. reported in an earlier study that railroad workers, heavy equipment operators, miners and truck drivers have higher mortality both for all causes and for lung cancer when compared with workers without exposure to DE [2]. Similar findings were also reported by Garshick *et al*. [11,13] and Larkin *et al*. [12]. However, a reanalysis of the US railroad study (originally published by Garshick [13]) indicates that the effect of DE exposure published in the early study appears to be unstable. The estimates of the effect vary strongly depending upon how the exposure was assessed and how confounders were considered in the analysis [14]. If the confounders were considered in a different manner, an exposure-response relationship between DE exposure and lung cancer is no longer observed. This early methodological disagreement in the US railroad study gives an example about how difficult previous evidence can be properly interpreted. This problem seems to be solved in a later published extended follow-up of this cohort [10]. Therefore, only the latest publication of this study [10] was considered in this review.

**Table 2:** Cohort studies on diesel exhaust exposure and lung cancer.

Author	Population	Follow-up time period	Exposure assessment	Confounder controlled	Statistical method	Job title/exposure	RR/SMR (95% CI)	Quantification of exposure doses
Ahlberg *et al*. (1981) [15]	35,960 drivers and 686,708 non-drivers	1961–1973	Job as professional driver	Age, sex, local region	Mantel-Haenszel	Driver	1.33 (1.13–1.56)	Impossible (exposure level and duration not available)
Attfield *et al*. (2012) [9]	12,315 non-metal miners	1947–1997	Historical measurement of CO	Age, Work location	SMR Cox-model	Highest expo. (≥1,280 µg/m^3^-year)	2.39 (0.82–6.94)	Possible (unit: µg/m^3^-year of respirable elemental carbon)
Balarajan *et al*. (1988) [16]	3,392 professional drivers in London	1950–1984	Job as professional driver in 1939	Age	SMR	Truck driver	1.59 (*p* < 0.05)	Impossible (exposure level and duration not available)
						Taxi driver	0.86 (*p* > 0.05)	
						Bus driver	1.42 (*p* > 0.05)	
Bender *et al*. (1989) [17]	4,849 highway maintenance workers	1945–1984	Job as highway maintenance worker	Age	SMR	Highway maintenance	0,69 (0.52–0.90)	Impossible (exposure level and duration not available)
Bergdahl *et al*. (2010) [7]	8,321 iron ore miners	1958–2000	100,000 historical measurement of NO_2_	Age and calendar period	SIR, Poisson regression	>15 (ppm-year)	0.87 (0.42–1.83)	Possible (unit: ppm-year of NO_2_)
Boffetta *et al*. (1988) [2]	461,981 males aged 40–79 years	1982–1984	Longest job with DME exposure	Age, smoking and other occupational exposures	Mantel-Haenszel	DE exposed	1.18 (0.97–1.44)	Impossible (exposure level not available)
						Truck driver	1.24 (0.93–1.66)	
						Railroad worker	1.59 (0.94–2.69)	
						Heavy equipment operator	2.60 (1.12–6.06)	
Boffetta *et al*. (2001) [18]	All Swedish population employed without farmer	1971–1989	Job titles 1960–1970, DME yes/no	Age	SIR, Poisson regression	DE low	0.95 (0.92–0.98)	Impossible (exposure level and duration not available)
						DE medium	1.1 (1.08–1.21)	
						DE high	1.3 (1.26–1.42)	
Garshick *et al*. (1988) [13]	55,407 US railroad workers	1959–1980	Job title in 1959 DME yes/no	Age	Cox-model	DE exposure (1–4 years)	1.20 (1.01–1.44)	Impossible (exposure level not available)
						DE exposure (5–9 years)	1.24 (1.06–1.44)	
						DE exposure (10–14 years)	1.32 (1.13–1.56)	
						DE exposure (≥15 years)	1.82 (1.30–2.55)	
Garshick *et al*. (2004) [19]	54,973 US railroad workers	1959–1996	Job title in 1959 DME yes/no	Age, year of employment	Cox-model	DE exposed	1.40 (1.30–1.51)	Impossible (exposure level not available)
Garshick *et al*. (2006) [10]	39,388 US railroad workers	1959–1996	Job title in 1959 DME yes/no	Age, Smoking	Cox-model	DE exposed	1.22 (1.12–1.32)	Impossible (exposure level not available)
						Conductor (<5 years)	1.31 (1.12–1.51)	
						Conductor (5–10 years)	1.23 (1.0–1.39)	
						Conductor (10–15 years)	1.23 (1.08–1.39)	
						Conductor (15–20 years)	1.16 (1.03–1.30)	
						Conductor (≥20 years)	1.22 (1.02–1.47)	
Garshick *et al*. (2008) [11]	31,135 truck industry workers	1985–2000	Job title (ever employed ≥ 1 year)	Age, race, smoking, healthy worker effect	Cox-model	Long-haul driver (20 years)	1.40 (0.88–2.24)	Impossible (exposure level not available)
						Pickup driver (20 years)	2.21 (1.38–3.52)	
						Dockworker (20 years)	2.02 (1.23–3.33)	
						Combination (20 years)	2.34 (1.42–3.83)	
Guberan *et al*. (1992) [20]	6,630 professional drivers	1949–1986	Job documen-ted as profess-ional driver	Age	SMR (SIR)	Driver	1.50 (1.23–1.81)	Impossible (exposure level and duration not available)
Guo *et al*. (2004) [6]	All economically active Finns on 31 December 1970 (*n* = 1,180,231)	1971–1995	Work history documented in Population Census File, FIN-JEM (historical measurement of NO_2_)	Smoking, asbestos, silica and socio-economic status	Poisson regression	DE low (0.1–1.9)	0.98 (0.94–1.03)	Possible (unit: mg/m^3^-year)
						DE middle (2.0–9.9)	1.04 (0.94–1.03)	
						DE high (≥10)	0.95 (0.94–1.03)	
Gustafsson *et al*. (1986) [21]	6,071 Swedishdock workers	1961–1980	Job as dock worker	Age	SMR (SIR)	Dock worker	1.29 (1.02–1.63)	Impossible (exposure level and duration not available)
Haldorsen *et al*. (2004) [22]	All Norwegians in 1970, age: 25-64	1971–1991	Job title	Age, smoking	SIR	Driver	1.58 (1.5–1.7)	Impossible (exposure level and duration not available)
						Engine/motor operator workers	1.34 (1.2–1.5)	
Hansen (1993) [23]	14,225 truck drivers	1970–1980	Self-reported job as truck driverin 1970	Age	SMR	Truck driver	1.6 (1.28–1.98)	Impossible (exposure level and duration not available)
Howe *et al*. (1983) [24]	43,826 retired railway workers	1965–1977	Job at time of retirement, DME yes/no	Age	SMR	DE probably exposed	1.35 (*p* < 0.001)	Impossible (exposure level and duration not available)
Jakobsson *et al*. (1997) [25]	96,438 professional drivers in Sweden	1971–1984	Job in 1970	Age, smoking (indirect adjustment)	SMR	Taxi driver	1.2 (1.0–1.4)	Impossible (exposure level and duration not available)
						Long-distance lorry driver	1.1 (0.9–1.3)	
						Short-distance lorry driver	1.2 (1.0–1.7)	
Järvholm *et al*. (2003) [26]	20,728 drivers and 119,984 carpenters/electricians	1971–1995	Job documented in health examination	Age	SMR (SIR)	Equipment operator	0.76 (0.58–0.97)	Impossible (exposure level and duration not available)
						Truck driver	1.14 (0.87–1.46)	
Johnston *et al*. (1997) [5]	18,166 British coalminers	1969–1992	historical measurement of NO, NO_2_	Age, smoking	Cox-model	Risk/unit exposure	1.23 (1.0–1.5)	Possible (unit: g/m^3^-hour)
Kaplan (1959) [27]	6,506 deceased railroad workers in US	1953–1958	Job documented in medical record	Age	SMR	Railroad worker	0.88 (0.65–1.16)	Impossible (exposure level and duration not available)
Laden *et al*. (2007) [28]	54,319 male employees in US	1985–2000	Job title	Age	SMR	Driver	1.1 (1.02–1.19)	Impossible (exposure level and duration not available)
						Dockworker	1.1 (0.94–1.30)	
Lagorio *et al*. (1992) [29]	1,446 workers of gasoline filling station	1981–1991	Employment duration	Age	SMR	Filling station worker	1.06 (0.64–1.65)	Impossible (exposure level not available)
Larkin *et al*. (2000) [12]	55,395 US railroad workers	195–1976	Job title in 1959 DME yes/no	Age, smoking	Poisson regression	Engineer/fireman	1.17 (0.79–1.74)	Impossible (exposure level not available)
						Brakemen/conductor	1.08 (0.76–1.54)	
						Shop worker	1.21 (0.80–1.83)	
Luepker *et al*. (1978) [30]	184,435 truck drivers	3 months in 1976	Union membership	Age	SMR	Truck driver	1.21 (*p* > 0.05)	Impossible (exposure level and duration not available)
Magnani *et al*. (1988) [31]	All population in England and Wales	1971–1971	Decennial JEM for death cases, estimation of risk set	Age social class	SMR	DE low	0.98	Impossible (exposure level and duration not available)
						DE middle	0.95	
						DE high	0.96	
Maizlish *et al*. (1988) [32]	1,570 deceased highway workers	1970–1983	CalTRANS employees	Age	PMR	Highway worker	0.98 (0.80–1.19)	Impossible (exposure level and duration not available)
Menck and Henderson (1976) [33]	Estimated population at risk in 1971 in Los Angeles	1968–1973	Job documented in death certificates	Age	SMR	Taxi driver	3.44	Impossible (exposure level and duration not available)
						Truck driver	1.65	
						Auto repair	1.46	
						Transportation	1.27	
Milham (1983) [34]	429,926 male and 25,066 female deaths	1950–1979	Job during most of lifetime	Age	PMR	Railroad worker	1.2	Impossible (exposure level and duration not available)
						Machine operator	1.4	
Netterstrom (1988) [35]	2,465 bus drivers	1978–1984	Job in 1978	Age	SMR	Bus driver	0.55 (0.33–0.99)	Impossible (exposure level and duration not available)
Neumeyer-Gromen *et al*. (2009) [8]	5,862 potash miners	1970–2001	255 measurement of TC value in 1992	Age, smoking	SMR Poisson regression, Cox-model	DE exposure (<1.29)	1.0	Yes (unit: mg/m^3^-year)
Säverin *et al*. (1999) [36]						DE exposure (1.26–2.04)	1.13 (0.46–2.75)	
						DE exposure (2.04–2.73)	2.47 (1.02–6.02)	
						DE exposure (2.73–3.90)	1.50 (0.56–4.04)	
						DE exposure (>3.90)	2.28 (0.87–5.97)	
Nokso-Koivisto and Pukkala (1994) [37]	8,391 locomotive drivers	1953–1991	Member of association	Age	SIR	Locomotive driver	0.86 (0.75–0.97)	Impossible (exposure level and duration not available)
Paradis *et al*. (1989) [38]	2,134 bus drivers	1962–1985	Job in payroll	Age	SMR	Bus driver	1.01 (0.70–1.38)	Impossible (exposure level and duration not available)
Pukkala *et al*. (1983) [39]	All population in Finland, (age: 35–69)	1971–1975	Job in 1970	Age	SIR	Railway driver	0.58 (*p* > 0.05)	Impossible (exposure level and duration not available)
						Road transport	1.06 (*p* > 0.05)	
Raffle (1957) [40]	London transport male staff	1950–1953	Job in 1950	Age	SMR	Bus driver	1,4 (0.94–2.0)	Impossible (exposure level and duration not available)
Raffnson (1988) [41]	295 marine engineers und 182 machinists	1955–1982	Job documented in the Register of Engineers	Age	SMR	Marine engineer	2.05 (0.83–4.23)	Impossible (exposure level and duration not available)
Rafnsson and Gunnarsdottir (1991) [42]	888 truck drivers and 726 taxi drivers alive in 1951	1951–1988	Job documented in truck driver union	Age	SMR	Truck driver	2.14 (1.37–3.18)	Impossible (exposure level and duration not available)
						Taxi driver	1.39 (0.72–2.43)	
Rushton *et al*. (1983) [43]	8,490 transport maintenance workers	1967–1975	Last or present job documented	Age	SMR	Maintenance Worker	1.01 (0.82–1.22)	Impossible (exposure level and duration not available)
Schenker (1984) [44]	2,519 railroad workers	1967–1979	Job title in retirement board, DME: Yes/No	Age	SMR	DE exposed	1.42 (0.92–1.92)	Impossible (exposure level and duration not available)
Stern *et al*. (1981) [45]	1,558 motor vehicle examiners	1944–1977	Ever employed job	Age	SMR	Motor vehicle examiner	1.02 (0.6–2.0)	Impossible (exposure level and duration not available)
Stern *et al*. (1997) [46]	Death of 15,843 construction operating engineers	1988–1993	Job title	Age	PMR	construction operating engineers	1.14 (1.09–1.19)	Impossible (exposure level and duration not available)
Waller (1981) [47]	Transport workers in London 420,699 man-years at risk	1950–1974	Job in 1950	Age	SMR	Bus driver	0.79 (0.73–0.85)	Impossible (exposure level and duration not available)
Waxweiler (1973) [48]	4,944 potash miners, US	1940–1967	Ever employed in a potash firm	Age	SMR	Potash miner	1.1 (0.69–1.66)	Impossible (exposure level and duration not available)
Wong *et al*. (1985) [49]	34,156 construction workers in US	1964–1978	Heavy equipment operators ≥20 year, duration of union membership	Age	SMR	Union membership	1,07 (1.00–1.15)	Impossible (exposure level not available)

Among the three cohort studies employing historical measurements of nitro compounds as surrogate indicators of DE exposures [5,6,7], a weak association (OR = 1.23, 95% CI: 1.0–1.5) between DE exposure and lung cancer can be demonstrated only in the study by Johnston *et al*. [5]. In the other two cohort studies [6,7], no relationship between DE exposure and lung cancer could be observed. Main strengths of these studies are large sample size, quantitative exposure estimations and consideration of smoking as a confounder in the analysis. However, some important limitations make the interpretation of these studies difficult. These include the population based setting and incomplete assessment of work history in the study by Guo *et al*. [6], and the missing consideration of occupational cofounders (such as respirable silica) in the analysis of the other two mining cohorts [5,7]. Since it is generally questionable if nitro compounds can be used as surrogate to measure DE exposures, the evidences provided by these studies are rather limited.

The German potash miner study [8] is the first study which quantified DE exposures by measuring carbon compounds. This study has a sample size of 5,862 workers with a follow-up duration of 30 years. After adjustment for age and smoking, the study demonstrates a clear exposure-response relationship between DE exposures and lung cancer mortality. However, in a recent reanalysis of this study, Möhner *et al*. [50] pointed out that a part of cohort members in this study were previously employed as uranium miners. These workers may have had a high exposure to respirable silica and radon daughters in their work history. If these subjects were excluded from the data analysis, an exposure-response relationship between DE exposure and lung cancer can no longer be observed. This finding leads to a further reanalysis of this cohort in which employment in external mines or industries was controlled [51]. The final results give no evidence of an association between DE exposure and lung cancer. Strengths of this study are large sample size and extensive control of both occupational and non-occupational confounders in the analysis [50,51]. Historical DE exposures were estimated based on the current industrial hygiene measurements.

In contrast to the German potash miner study, the US Miners study demonstrates an extremely high effect of DE exposure (up to 5-fold), although the initial analysis of this cohort did not reveal a clear relationship between DE exposure and lung cancer [9]. Main strengths of this study are large sample size (more than 12,000 workers with an average follow-up duration of about 23 years), quantitative assessment of DE exposures by measuring carbon compounds and the adjustment of smoking as a confounder in a nested case-control analysis [52]. However, some findings reported in this study need more clarification. For example, it is unclear why “surface only workers” (SMR = 1.33) have the same risk as the “ever underground workers” (SMR = 1.21) in the initial analysis, although DE exposure among “underground workers” was about 500 times higher than “surface workers”. This finding seems to be contradictory with the final reported high effect of DE exposures. Possible limitations of this study have been discussed by Morfeld [53] and Gamble *et al*. [54] regarding the completeness of follow-up, essential exposure misclassification, inadequate control of occupational confounder and improper statistical methods used.

**Figure 1:**
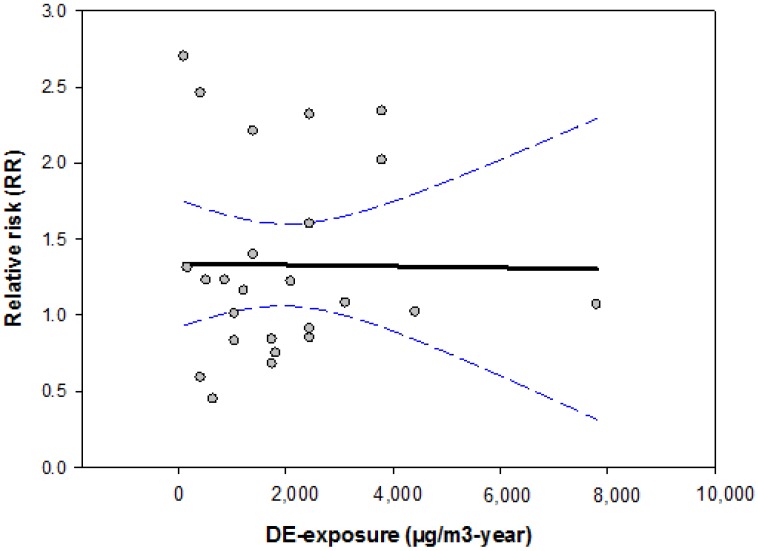
Effects of DE-exposures on the risk of lung cancer given in previously published cohort studies.

In order to compare previously published cohort studies objectively and to allow an overall judgement of the association between DE exposure and lung cancer, we calculated the historical DE exposure in previous studies by means of the MEGA-JEM. Due to limited exposure information (limited information on job title or exposure duration), cumulative doses of DE exposures are only available for six cohort studies (Table S1, Supplementary Information). The results of these studies are summarized in Figure 1. Overall, no exposure-response relationship between DE exposure and lung cancer can be demonstrated.

### 3.2. Case-Control Studies

In total, 25 population or hospital-based case-control studies, six nested case-control studies and 1 industry-based case-referent study were identified (see Table 3). Most of these studies have large sample sizes and adjustment of the possible confounding effect of smoking in the analysis.

Assessments of DE exposures were limited in most of these studies on job title (with different definitions) or dichotomous categorization (ever/never exposed). Quantitative or semi-quantitative assessment of DE exposure was carried out in only six studies, with use of different exposure assessment methods [51,52,55,56,57,58]. Overall, a consistently increased risk of lung cancer was reported for jobs supposed to have high DE exposures. An exposure-response relationship was also presented in most studies. However, due to the different exposure assessment methods used, direct comparison between these studies is difficult.

**Table 3:** Case-control studies on diesel exhaust exposure and lung cancer.

Author	Design	Population	Exposure assessment	Confounder controlled	Statistical method	Job title/exposure	OR	Quantification of exposure doses
							(95% CI)	
Benhamou *et al*. (1988) [59]	Population based case-control study	1,625 cases and 3,091 controls	Ever employed as professional driver	Age, smoking	Conditional logistic regression	Motor vehicle driver	1.42 (1.07–1.89)	Impossible (exposure level and duration not available)
						Transport equipment operator	1.35 (1.05–1.75)	
						Miner	2.14 (1.07–4.31)	
						Farmers	1.24 (0.94–1.62)	
Boffetta *et al*. (1990) [60]	Population based case-control study	2,584 cases and 5,099 controls	Self reported exposure (yes/no)	Age, race, smoking, education and asbestos	Logistic regression	Probable DE exposure	1,49 (0,72–3,11)	Impossible (exposure level not available)
						(≥30 years)		
						Truck driver	1,83 (0,31–10,73)	
						(1–15 years)		
						Truck driver	0,94 (0,41–2,15)	
						(16–30 years)		
						Truck driver	1,17 (0,40–3,41)	
						(>30 years)		
Brüske-Hohlfeld *et al*. (1999) [61]	Population based case-control study	3,498 cases and 3,541 controls	Interview on work history	Age, smoking and Asbestos	Conditional logistic regression	DE exposed	1,43 (1,23–1,67)	Impossible (exposure level and duration not available)
Buiatti *et al*. (1985) [62]	Population based case-control study	376 cases and 892 controls	Ever employed job transportation	Age and smoking	Logistic regression	Transportation	1.1 (0.7–1.6)	Impossible (exposure level and duration not available)
						Taxi driving	1.8 (1.0–3.4)	
						Train conductor	1.4 (0.5–3.9)	
Burns (1991) [63]	Population based case-control study	5,935 cases and 3,956 controls with colon cancer	Telephone interview on work history, job title	Age and smoking	Logistic regression	Automobile repair	1.56 (0.85–2.87)	Impossible (exposure level and duration not available)
						Railroad	1.37 (0.70–2.66)	
						Bus and truck transport	1.20 (0.82–1.75)	
Coggon *et al*. (1984) [64]	Population based case-control study	598 cases and 1,180 controls	Job in death certificate DME (yes/no)	Age, sex and residence	Logistic regression	High DE jobs	1.1 (0.7–1.8)	Impossible (exposure level and duration not available)
Damber and Larsson (1987) [65]	Population based case-control study	589 cases and 1,035 controls	Self reported work history	Age and smoking	Logistic regression	Professional driver	1.36 (0.97–1.91)	Impossible (exposure level not available)
						(>1 years)		
						Professional driver	1.47 (0.97–2.20)	
						(>10 years)		
						Professional driver	1.61 (1.01–2.57)	
						(>20 years)		
Decoufle *et al*. (1977) [66]	Hospital based case-control study	Cases and controls were selected among 13,949 patients	Job title	Age and smoking	unclear	Bus driver	1.81 (*p* < 0.05)	Impossible (exposure level and duration not available)
						Taxi driver	0.82 (*p* < 0.05)	
						Truck driver	1.07 (*p* < 0.05)	
Elci *et al*. (2003) [67]	Hospital based case-control study	1,354 cases and 1,519 controls	Job title	Age and smoking	Logistic regression	Driver	1.4 (1.1–2.0)	Impossible (exposure level and duration not available)
						Highway construction	1.5 (1.1–2.5)	
Emmelin (1993) [55]	Industry based case-referent study	50 cases and 154 controls (dock workers)	Job as dock worker. Index for DME exposure	Age and smoking	Conditional logistic regression	Low DE	reference	Impossible (exposure level and duration not available)
						Medium DE	1.6 (0.5–5.1)	
						High DE	2.9 (0.8–10.7)	
Garshick *et al*. (1987) [68]	Nested case-control study	Deceased railroad workers. 1,256 cases and 2,385 controls	Expert evaluation for jobs, exposure duration	Age, smoking and asbestos	Logistic regression	Railroad	1.55 (1.09–2.21)	Impossible (exposure level not available)
						(>20 years)		
						DE exposed	1.41 (1.06–1.88)	
						(>20 years)		
Gustavsson *et al*. (1990) [56]	Nested case-control study	20 cases and 120 controls	Index for exposure level, exposure duration	Age and asbestos	Conditional logistic regression	Index value 1	Reference	Impossible (exposure level and duration not available)
						(0–10)		
						Index value 2	1.34 (1.09–1.64)	
						(10–20)		
						Index value 3	1.81 (1.20–2.71)	
						(20–30)		
						Index value 4 (>30)	2.43 (1.32–4.47)	
Gustavsson *et al*. (2000) [57]	Population based case-referent study	1,042 cases and 1,274 controls	historical measurement of NO_2_	Age, smoking, radon	Logistic regression	0–0.53	0.67 (0.42–1.08)	DME was calculated as cumulative NO_2_ exposure (mg/m^3^-year)
						0.54–1.41	1.14 (0.77–1.67)	
						1.42–2.37	1.01 (0.67–1.53)	
						≥2.38	1.62 (1.13–2.31)	
Hall *et al*. (1984) [69]	Hospital based case-control study	502 cases and 502 controls	Interview on job title	Age, smoking and social status	Mantel-Haenszel	Bus driver	5.5 (0.8–36.0)	Impossible (exposure level and duration not available)
						Truck driver	1.4 (0.7–2.6)	
						Railroad worker	2.6 (0.5–12.8)	
						Heavy equipment	3.5 (1.0–11.8)	
Hansen *et al*. (1998) [70]	Population based case-control study	37,597 cases and 37,597 controls	Job title documented in National Bureau of Statistics	Age and sex	Conditional logistic regression	Taxi driver	1.6 (1.2–2.2)	Impossible (exposure level and duration are not available)
						Bus and truck driver	1.3 (1.2–1.5)	
Hayes *et al*. (1989) [71]	Population based case-control study	1,444 cases and 1,893 controls	Interview, motor exhaust-related jobs, employment duration	Age, smoking and study area	Logistic regression	Truck driver	1.5 (1.1–1.9)	Impossible (exposure level not available)
						(≥10 years)		
						Bus driver	1.6 (0.9–2.8)	
						(≥10 years)		
						Mechanics	1.7 (0.9–3.4)	
						(≥10 years)		
						Heavy equipment	1.3 (0.6–3.1)	
						(≥10 years)		
Kauppinen (1993) [72]	Nested case-control study	136 cases and 408 controls	JEM for job title, DME (yes/no)	Age, smoking	Conditional logistic regression	DE exposed	1.70 (0.55–5.20)	Impossible (exposure level and duration not available)
Lerchen *et al*. (1987) [73]	Population based case-control study	506 cases and 771 controls	High risk jobs ever exposed?	Age, sex, race and smoking	Logistic regression	Engineer and fireman	0.6 (0.1–3.3)	Impossible (exposure level and duration not available)
						Diesel engine mechanic	0.6 (0.2–2.0)	
						ME exposure	0.6 (0.2–1.6)	
Milne *et al*. (1983) [74]	Population based case-control study	925 cases and 6,420 cancer controls	Job title in death certificates	Age and sex	Logistic regression	Transportation	1.1	Impossible (exposure level and duration not available)
Möhner *et al*. (2013) [51]	Nested case-control study	68 cases and 340 controls	255 measurement of TC value in 1992	Age, smoking, external employment	Conditional logistic regression	1st quartile	reference	Yes (unit: μg/m^3^-year)
						2nd quartile	0.90	
						3rd quartile	1.16	
						4th quartile	0.78	
Olsson *et al*. (2011) [75]	Pooled analysis of 11 case-control studies	13,304 population cases and 16,282 controls			Logistic regression	Exposure index > 34.5	1.31 (1.19–1.43)	Impossible (exposure level not available)
Parent *et al*. (2007) [76]	Population based case-control study	857 cases and 1,882 controls			Logistic regression	DE exposure	1.2 (0.8–1.8)	Impossible (exposure level not available)
Pfluger and Minder (1994) [77]	Population based case-control study	Deceased chauffeurs	Job title in death certificates	Age and smoking	Poisson regression	Chauffeur	1.48 (1.30–1.68)	Impossible (exposure level and duration not available)
Richiardi *et al*. (2006) [78]	Population based case-control study	595 cases and 845 controls	Job title, DME (yes/no)	Age, sex, smoking and other occupational exposures	Logistic regression	DE exposure	1.04 (0.79–1.37)	Impossible (exposure level and duration not available)
Siemiatycki *et al*. (1988) [79]	Hospital based case-control study	857 cases and 1,523 controls	Interview on work history, expert judgement on DE exposure	Age, race, social status, smoking and blue/white collar job	Mantel-Haenszel	DE exposed	1.2 (0.8–1.5)	Impossible (exposure level and duration not available)
Silverman *et al*. (2012) [52]	Nested case-control study	198 cases and 562 controls from 8 mining companies	1,156 measurement of EC value during 1998–2001	Age, sex, race, smokig and history of respiratory disease	Conditional logistic regression	DE exposure	Reference	Yes (unit: μg/m^3^-year)
						(0–19)		
						DE exposure	0.87 (0.48–1.59)	
						(19–246)		
						DE exposure	1.50 (0.67–3.36)	
						(246–964)		
						DE exposure	1.75 (0.77–3.97)	
						(≥964)		
Soll-Johanning *et al*. (2003) [80]	Nested case-control study	153 cases and 606 controls	Job as bus driver	Age and smoking	Conditional logistic regression	20+ years of employment	0.63 (0.32–1.14)	Impossible (exposure level not available)
Steenland *et al*. (1990) [81]	Population based case-control study	996 cases and 1,085 controls	Interview next of kin, longest job as truck driver	Age, smoking and asbestos	Multivariate analysis	Truck driver	1.55 (0.97–2.47)	Impossible (exposure level not available)
						(≥18 year)		
						Truck mechanic	1.50 (0.59–3.40)	
						(≥18 year)		
Swanson *et al*. (1993) [82]	Population based case-control study	3,797 cases and 1,966 controls (colon cancer)	Interview relatives, last job title, employment duration	Age, race and smoking	Logistic regression	Industrial maintenance (20+ years)	1.5 (0.8–2.9)	Impossible (exposure level not available)
						Automobile mechanics (20+ years)	1.5 (0.7–3.0)	
						Machine operators (20+ years)	1.9 (1.0–3.9)	
						Heavy truck driver (20+ years)	2.5 (1.4–4.4)	
						Light truck driver (20+ years)	2.1 (0.9–4.6)	
Villeneuve *et al*. (2011) [58]	Population based case-control study	1,681 cases and 2,053 controls	Expert evaluation for jobs	Age, smoking, location, silica and asbestos	Logistic regression	Cumul. expo. 1. tertile	0.93 (0.75–1.17)	Impossible (exposure level and duration not available)
						Cumul. expo. 2. tertile	1.03 (0.83–1.29)	
						Cumul. expo. 3. tertile	1.12 (0.89–1.40)	
Wegman and Peters (1978) [83]	Population based case-control study	100 cases and 100 controls of CNS cancer	Tele. Interview relatives on job title	No	Logistic regression	Transportation equipment operator	1.26 (0.28–5.84)	Impossible (exposure level and duration not available)

**Figure 2:**
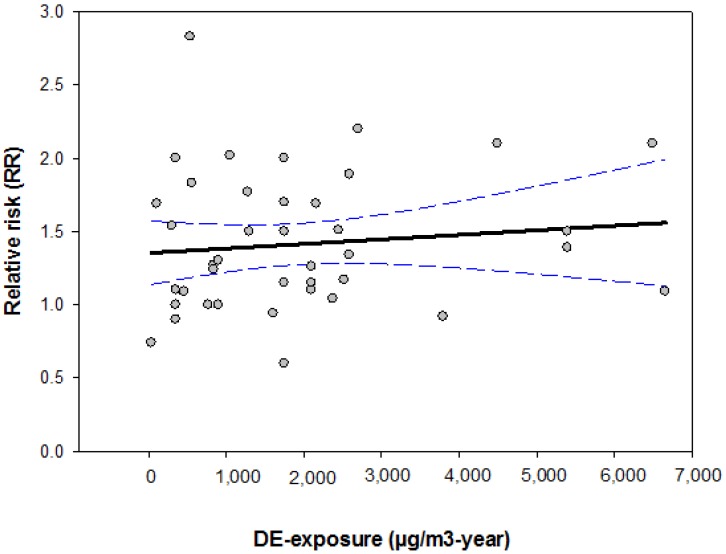
Effects of DE-exposures on the risk of lung cancer given in previously published case-control studies.

To facilitate the comparison of previously published case-control studies, we assessed the DE exposure quantitatively by means of the MEGA-JEM. Due to limited exposure information, cumulative doses of DE-exposures can only be quantified for eight case-control studies (Table S2, Supplementary Information). The results of these studies are summarized in Figure 2. Similar to previously published cohort studies, case-control studies do not show a clear exposure-response-relationship.

## 4. Discussion

The possible association between DE and lung cancer, which constitutes an important occupational health question, has long been the subject of debate. Interpretation of epidemiological evidence faces a series of methodological challenges.

Lack of exposure information appears to be the major problem in interpreting human epidemiological data. The low volume of data documenting past exposures is due to the fact that no standardized method of measuring diesel fumes existed before the late 1980s. From an industrial hygiene prospective, it was not clear which substance to measure during assessment of occupational exposure to DE. Diesel fumes are composed of gases (nitrogen oxides, carbon monoxide) and various hydrocarbons bound to a carbon core. Early studies have reported levels of particulate, but such particulates are generated by many sources other than diesel engines [84]. Attention has also been focused on polycyclic aromatic hydrocarbons (PAHs) and nitro-PAHs in the exhaust. However, there are no standard methods of measuring PAHs, and PAHs are also emitted by sources other than diesel engines [84].

In the late 1980s, a standardized method of measuring diesel fumes by quantifying elemental carbon was introduced. Since then, systematic industrial hygiene measurements have been begun in some industrialized countries. However, a long time is needed for sufficient measurement data to be collected for use in epidemiological research. Most of the epidemiological studies published to date therefore provide no fundamental basis for an objective assessment of DE exposures.

In this review, we identified only two recent studies containing industrial hygiene measurement data for carbon compounds. In all remaining studies, the exposure assessments are based on expert judgements. A given job may be classified as having high exposure by one expert, but low by another [14,85]. Previous studies indicate that the differences in expert opinion have a strong influence on the estimated exposure-response relationship between DE exposure and lung cancer [14,85]. This problem makes the interpretation and comparison of previously published epidemiological studies difficult.

To facilitate an objective comparison of previously published epidemiological studies, we created a JEM for DE exposures based upon a large number of standardized industrial hygiene measurements conducted since the late 1980s. Three calendar periods were considered in the JEM, since most of the technical changes occurred during the period between 1990 and 1993. The values in the MEGA-JEM were considered in the interpretation of the epidemiological studies published to date. We found that conflicting findings were reported not only between studies, but also within studies. It is very common for jobs associated with higher exposure (according to the exposure value given in Table 1) to be reported as having lower risks than jobs with lower exposure, even within the same study. Since many studies indicated only job titles without detailed information on the exposure duration, direct comparison of the effect estimates was limited. To solve this problem, we summarized only studies with complete exposure information (both job title and exposure duration) and presented the results in Figure 1 and Figure 2. Overall, neither cohort nor case-control-studies show exposure-response relationship between DE exposure and lung cancer. 

Caution should be exercised during interpretation of these studies. Previous cohort studies often compare workers in certain job categories with a standard population without adjustment for important confounders, while case-control studies generally employ a population-based design which is less suitable for detecting weak associations related to DE exposures. For some of the early epidemiological studies, latency may also be too short to attribute lung cancer to DE exposure. The use of different definitions of job titles in the analysis (longest job, ever employed jobs, census job, job in death certificates or at the time of medical examination, *etc*.) and the related cross-contamination with current and previous occupational history may also have a strong influence on the estimated effects. This problem was clearly demonstrated in the cohort of German potash miners, for which the study results were strongly dependent upon whether previous work history in the uranium mining industry was considered in the analysis [50]. The JEM-approach used in this review has also some weaknesses. First, the exposure duration in most studies is given only in categories. Therefore, the use of the center of such category gave only a very crude estimate for the mean or the median of exposure duration. Furthermore, the JEM used in this review is based on German industrial hygiene measurement data. The data collected in Germany may not be representative for all industrialized countries. Since diesel engines were introduced into the workplace at variable rates over time by industry and country, the use of MEGA-JEM in this review may lead to some uncertainty in the exposure assessment. However, despite the exposure-assessment methods used (expert judgement, measuring nitro compounds, measuring carbon compound, MEGA-JEM) no consistent findings of an association between DE exposures and lung cancer can be demonstrated.

## 5. Conclusions

Overall, the previously published epidemiological evidence did not clearly support an exposure-response relationship between DE exposure and lung cancer. In fact, the limited exposure information available in previous studies does not even allow a valid estimation of an association between DE exposure and lung cancer. However, such an association cannot be ruled out. Causality of weak association is often difficult to establish, since it is susceptible to all forms of possible design bias. Due to the limited epidemiological evidence to date, well designed studies in an industrial context are still needed, for which detailed exposure assessment methods and adequate control for confounders are recommended.

## Supplementary Files

Supplementary File 1 Supplementary Information (PDF, 126 KB)
