# Supplementary material for: Diesel Exhaust Exposure and the Risk of Lung Cancer—A Review of the Epidemiological Evidence
Source: Int J Environ Res Public Health. 2014 Jan 27;11(2):1312–40. doi: 10.3390/ijerph110201312 (PMC3945540; doi:10.3390/ijerph110201312)
Supplement: Supplementary File 1 — Supplementary Information (PDF, 126 KB) [file ijerph-11-01312-s001.pdf]

Diesel Exhaust Exposure and the Risk of Lung Cancer—A Review of the Epidemiological Evidence

Table S1. Data points estimated for Figure 1 (cohort studies).

Study	Job title	Exposure * level (μg/m^3)	Exposure ** duration (Y)	Cumulative doses (μg/m^3-Y)	RR
Garshick [1]	Engineer/Conductor	70	2.5	175	1.31
			7.5	525	1.23
			12.5	875	1.23
			17.5	1,225	1.16
			30	2,100	1.22
Garshick [2]	Long-haul driver	70	20	1,400	1.4
	Pickup driver	70	20	1,400	2.21
	Dockworker	190	20	3,800	2.02
	Combination workers	190	20	3,800	2.34
Rafnsson [3]	Truck driver	70	1.5	105	2.7
			6	420	2.46
			25	1,750	0.68
			38	2,450	2.32
	Taxi driver	70	69	42	0.59
			35	2,450	1.6
Schenker [4]	Railway workers	70	15	1,050	0.83
			25	1,750	0.84
			35	2,450	0.91
Wong [5]	Heavy equipment operator	260	2.5	650	0.45
			7	1,820	0.75
			12	3,120	1.08
			17	4,420	1.02
			30	7,800	1.07
Paradis [6]	Bus driver	70	15	1,050	1.01
			35	2,100	0.85

Notes: * Exposure levels estimated according to MEGA-JEM; ** Exposure information given by published studies.

Table S2. Data points estimated for Figure 2 (case-control studies).

**Study**	**Job title**	**Exposure * level (µg/m³)**	**Exposure ** duration (Y)**	**Cumulative doses (µg/m³-Y)**	**RR**
Boffetta [7]	Truck driver	70	8	560	1.83
			23	1,610	0.94
			36	2,520	1.17
Brüske-Hohlfeld [8]	Professional driver	70	1.5	105	1.69
			6.5	455	1.09
			15	1,050	2.02
			25	1,750	1.15
			35	2,450	1.51
Damber [9]	Driver	70	11	770	1.0
			30	2,100	1.1
Hayes [10]	Truck driver	70	5	350	1.0
			25	1,750	1.5
	Heavy equipment operator	260	5	1,300	1.5
			25	6,500	2.1
	Bus driver	70	5	350	1.1
			25	1,750	1.7
	Taxi driver	70	5	350	2.0
			25	1,750	2.0
	Other Driver	70	5	350	0.9
			25	1,750	0.6
	Mechanics	180	5	900	1.0
			25	4,500	2.1
Steenland [11]	Diesel truck driver	70	12	840	1.27
			30	2,100	1.26
			37	2,590	1.89
	Gasoline truck driver	70	12	840	1.24
			30	2,100	1.1
			37	2,590	1.34
	Both truck driver	70	12	840	1.24
			30	2,100	1.15
			37	2,590	1.34
	Mechanics	180	12	2,160	1.69
			30	5,400	1.39
			37	6,660	1.09
	Dock workers	190	20	3,800	0.92
Swanson [12]	Mechanics	180	5	900	1.3
			15	2,700	2.2
			30	5,400	1.5
Silverman [13] **				37.5	0.74
				304	1.54
				536	2.83
Möhner [14] **				1,279	1.77
				2,375	1.04

Notes: * Exposure levels estimated according to MEGA-JEM; ** Exposure information given by published studies.

References

1. Garshick, E.; Laden, F.; Hart, J.E.; Smith, T.J.; Rosner, B. Smoking imputation and lung cancer in railroad workers exposed to diesel exhaust. *Amer. J. Ind. Med.* **2006**, *49*, 709–718.
2. Garshick, E.; Laden, F.; Hart, J.E.; Rosner, B.; Davis, M.E.; Eisen, E.A.; Smith, T.J. Lung cancer and vehicle exhaust in trucking industry workers. *Environ. Health Perspect.* **2008**, *116*, 1327–1332.
3. Rafnsson, V.; Gunnarsdottir, H. Mortality among professional drivers. *Scand. J. Work Environ. Health* **1991**, *17*, 312–317.
4. Schenker, M.B.; Smith, R.; Munoz, A.; Woskie, S.; Spezier, F.E. Diesel exposure and mortality among railroad workers: Results of a pilot study. *Brit. J. Ind. Med.* **1984**, *41*, 320–327.
5. Wong, O.; Morgan, R.K.; Kheifets, L.; Larson, S.R.; Whorton, M.D. Mortality among members of a heavy construction equipment operators union with potential exposure to diesel exhaust emission. *Brit. J. Ind. Med.* **1985**, *42*, 435–448.
6. Paradis, G.; Theriault, G.; Tremblay, C. Mortality in a historical cohort of bus drivers. *Int. J. Epidemiol.* **1989**, *18*, 397–402.
7. Boffetta, P.; Harris, R.E.; Wynder, E.L. Case-control study on occupational exposure to diesel exhaust and lung cancer risk. *Amer. J. Ind. Med.* **1990**, *17*, 577–591.
8. Brüske-Hohlfeld, I.; Möhner, M.; Ahrens, W.; Pohlabeln, H.; Heinrich, J.; Kreuzer, M.; Jöckel, K.H.; Wichmann, H.E. Lung cancer risk in male workers occupationally exposed to diesel motor emissions in Germany. *Amer. J. Ind. Med.* **1999**, *36*, 405–414.
9. Damber, L.A.; Larsson, L.G. Occupation and male lung cancer: A case-control study in northern Sweden. *Brit. J. Ind. Med.* **1987**, *44*, 446–453.
10. Hayes, R.B.; Thomas, T.; Silverman, D.T.; Vineis, P.; Blot, W.J.; Mason, T.J.; Pickle, L.W.; Correa, P.; Fontham, E.T.; Schoenberg, J.B. Lung cancer in motor exhaust-related occupations. *Amer. J. Ind. Med.* **1989**, *16*, 685–695.
11. Steenland, N.K.; Silverman, D.T.; Hornung, R.W. Case-control study of lung cancer and truck driving in the Teamster Union. *Amer. J. Public Health* **1990**, *80*, 670–674.
12. Swanson, G.M.; Lin, C.S.; Burns, P.B. Diversity in the association between occupation and lung cancer among black and white men. *Cancer Epidemiol. Biomarkers Prev.* **1993**, *2*, 313–320.
13. Silverman, D.T.; Samanic, C.M.; Lubin, J.H.; Blair, A.E.; Stewart, P.A.; Vermeulen, R.; Coble, J.B.; Rothman, N.; Schleiff, P.L.; Travis, W.D.; *et al*. The diesel exhaust in miners study: A nested case-control study of lung cancer and diesel exhaust. *J. Nat. Cancer Inst.* **2012**, *104*, 855–868.
14. Möhner, M.; Kersten, N.; Gellissen, J. Diesel motor exhaust and lung cancer mortality: Reanalysis of a cohort study in potash miners. *Eur. J. Epidemiol.* **2013**, *28*, 159–168.

© 2014 by the authors; licensee MDPI, Basel, Switzerland. This article is an open access article distributed under the terms and conditions of the Creative Commons Attribution license (http://creativecommons.org/licenses/by/3.0/).
